# Social media stethoscope: unraveling how doctors’ social media behavior affects patient adherence and treatment outcome

**DOI:** 10.3389/fpubh.2024.1459536

**Published:** 2024-09-20

**Authors:** Qian Sun, Guiyao Tang, Wenxiao Xu, Shaoli Zhang

**Affiliations:** ^1^School of Management, Shandong University, Jinan, China; ^2^Shandong Institute of Talent Development Strategy, Shandong University, Jinan, China; ^3^The College of Business, University of Nevada, Reno, Reno, NV, United States; ^4^School and Hospital of Stomatology, Cheeloo College of Medicine, Shandong University, Jinan, China

**Keywords:** social media behavior of doctors, patient adherence, treatment outcome, gender bias, doctor humor

## Abstract

**Objective:**

The exposure of the content posted by doctors on social media has the potential to influence how patients perceive and judge doctors. It is necessary to further investigate whether and how the content posted by doctors affects patients’ health behaviors and outcomes, as well as to identify the factors that may influence this mechanism.

**Methods:**

Multi-respondent survey data was collected from 35 doctors and 322 patients in China, and structural equation modeling (SEM) was used to test the hypothesis model.

**Results:**

The findings revealed that doctors posting professional knowledge content on social media positively impacted patient adherence and treatment effectiveness. Conversely, doctors sharing personal life-related content on social media were associated with lower patient adherence and poorer treatment outcome. Moreover, doctor gender and doctor humor moderate the relationship between social media behavior of doctors and patient adherence.

**Conclusion:**

Doctors sharing professional knowledge on social media not only fosters trust in physicians but also closely correlates with patient adherence and treatment effectiveness.

## 1 Introduction

Social media, as Internet-based channels, provides users with the opportunity to interact and present themselves selectively (1). It encompasses diverse online forums that facilitate communication through various mediums such as audio, text, images, and videos. Given the immense user base of platforms like Facebook and X (formerly known as Twitter), social media offers a valuable opportunity for doctors and patients to engage and interact with each other (2). Dedicated forums, like MS Society UK and PatientsLikeMe, provide platforms where patients can share their experiences. Patients use these forums primarily for a variety of purposes, including expressing emotions, comparing their experiences with other members, and seeking information and network support (3, 4). Research indicates that such social media behavior can enhance patients’ self-management and control (3), contribute to preventive behaviors (5), and support health behavior change interventions (6). However, alongside their numerous benefits, these platforms also pose significant challenges related to the data privacy of patients (7). Patients may share sensitive information or engage in discussions about their health, often without fully understanding how their data might be used or protected. This highlights the need for robust privacy measures and clear guidelines to protect patient information while leveraging the benefits of digital interactions.

Besides patients, a growing number of doctors are turning to social media to collect and disseminate treatment information, collaborate with colleagues on patient-related matters (7), and interact directly with patients to improve clinical care (8). The social media behavior of doctors is a crucial component of their behavior patterns; whether it aligns with group stereotypes and established expectations can significantly influence how patients perceive and react to them (9). Fatollahi et al. (10) explored the impact of doctors’ social media content on patient trust, revealing that the nature of the content shared significantly affects how much patients trust their doctors. Wu et al. (2) proposed a framework distinguishing between instrumental and affective interactions in doctor-consumer social media interaction. Their research demonstrated that these two interaction types influence consumers’ health behaviors through declarative knowledge, self-efficacy, and outcome expectancy. This progression highlights how the type of content and the nature of interactions in social media collectively shape patient trust and health behaviors. However, it remains unclear whether the social media behavior exhibited by doctors has the potential to affect patient health outcomes, and what specific factors might mediate or moderate this influence.

In China, tertiary stomatological hospitals serve as high-end institutions within the dental healthcare system, striving for excellence not only in clinical treatment but also in utilizing social media as a key platform for communication, education, and health management with patients. Affected by the COVID-19 pandemic, several stomatological hospitals introduced WeChat consultation services in 2020, providing patients with free online Q&A. Following this, they launched official accounts on social media platforms such as TikTok to promote their institutions and share knowledge about oral care. As a result, an increasing number of doctors are becoming active on social media. Therefore, this research focused on the social media behaviors of physicians in tertiary stomatology hospitals and aimed to investigate the potential impact of various types of these behaviors on patient health outcomes.

A study reveals that over 60% of doctors utilize various forms of social media for either personal or professional purposes (11). Consequently, the social media behaviors exhibited by physicians can be neatly categorized into two distinct domains: professional utilization and personal utilization (12). The professional utilization of social media by doctors has been recognized as an effective approach to staying connected, advancing professional expertise, educating patients, and enhancing the reputation of the medical profession (12–14). By leveraging both asynchronous and synchronous communication capabilities of social media, doctors and patients can establish real-time connections and exchange vital information (15), thus enhancing the conventional healthcare service process (16). Therefore, such social media behavior has the potential to enhance patient compliance and treatment outcomes. On the other hand, the personal utilization of social media by doctors may blur the boundaries between the professional and personal lives of doctors (7) and divert patients’ attention away from the medical expertise and knowledge they expect from their healthcare providers (12). In addition, inappropriate personal content posted by doctors on social media can undermine patient confidence in the medical profession (13). If a doctor engages in posting racist comments online, writing disrespectful patient narratives, posting intoxication photographs, or writing profanity, it would lead to a decrease in patient trust (10). Compared with professional utilization, the personal utilization of social media will lead to a perception of unprofessionalism and raise concerns about the doctor’s ability to maintain confidentiality and objectivity, hindering patients’ adherence to the medical advice and recommendations provided by the doctor.

Patient adherence is a significant behavioral response of patients toward doctors, reflecting their willingness to adhere to medical instructions and recommendations (17), such as taking medication, following diets, or executing lifestyle changes. Furthermore, adherence goes beyond compliance, reflecting a commitment to active participation and shared decision-making (17). Patients who actively engage in their healthcare become valuable partners in the treatment process, increasing the likelihood of achieving more favorable treatment outcome (18). Therefore, this study selected patient adherence as the core variable to deepen the understanding of the relationship between the doctor-patient relationship, patient behavior, and medical outcomes.

What is more, demographic characteristics and individual traits may also play a role in shaping perceptions and responses. The gender role stereotypes elicit different patient reactions to similar social media behavior exhibited by male and female doctors. Prior research indicates that female doctors face more barriers in promotion and receive less respect than their male colleagues (19–21). While women have made significant progress in the field of medicine, male doctors continue to be regarded as more authoritative and more professional (22, 23). Consequently, whether female doctors post professional knowledge content or personal life content on social media, they may encounter more bias and skepticism compared to their male counterparts. Simultaneously, this study examined the moderating role of doctor humor. Doctor humor was defined as an action enacted by a doctor toward a patient that is intended to be amusing to a patient and that a patient interprets as an intentional action (24). Humor in the past was commonly regarded as a low form of behavior (25), and there exists a certain conflict between humor and doctors’ professional role. Poorly enacted or misperceived humor can lead to patient skepticism toward a doctor’s professional competence (26), and undermine patients’ trust in doctors sharing content on social media (12). Therefore, we infer that the humor of doctors will harm patient adherence, regardless of the content they post on social media.

The swift rise in social media use necessitates a thorough understanding of both the reasons behind doctors’ social media activity and the ways in which patients perceive and are influenced by this behavior. Therefore, this study aims to explore the intricate dynamics of doctor social media behavior and its impact on patient behavior and eventual treatment outcome, as well as the moderating role of doctor gender and doctor humor. By analyzing how doctors’ behavior on social media affects patient health behaviors and treatment outcomes, this study contributes to revealing the potential impact of social media interactions on patient engagement and treatment outcome. The findings could provide guidance on how doctors can effectively use social media to better support patient health management and treatment processes, thereby enhancing patient communication experiences and satisfaction. Overall, this study will offer empirical evidence for doctors and healthcare organizations to optimize their use of social media tools, ultimately improving patient health behaviors and treatment outcomes.

## 2 Method

### 2.1 Procedure

This study was conducted in a tertiary stomatological hospital in eastern China and was approved by the ethics committee of the hospital (No. 20221207). The stomatological hospital provides a specific environment characterized by close doctor-patient online interactions and the need for long-term healthcare. This character allows for the collection of data at multiple time points and facilitates the exploration of the relationship between doctor social media behavior, patients’ behavior, and treatment outcome. The whole data collection process took 3–4 months.

To minimize and manage bias in the survey process, several measures were implemented. The questionnaire was first pilot-tested with a small group of participants to identify and address any issues related to question clarity or potential bias. Random sampling methods were then employed to reduce selection bias and recruit a diverse sample of healthcare professionals (HCPs) and patients, ensuring a broad range of perspectives. Additionally, the survey was conducted anonymously to encourage honest responses, with participants assured that their responses would be kept confidential, thereby reducing social desirability bias and enhancing the accuracy of the data collected.

The recruitment of healthcare professionals (HCPs) for this survey was strategically designed to ensure a diverse and representative sample. We focused on HCPs from various specialties who are actively engaged in social media, aiming to capture a wide range of perspectives on social media behavior and its impact on patient interactions. As a result, we recruited 50 doctors from the Department of Oral and Maxillofacial Surgery, Department of Prosthodontics, Department of Orthodontics, and Department of Endodontics, who create original content on social media platforms such as Toutiao, Weibo, TikTok, and WeChat, and are willingly to participate in this study. The original content created by these doctors included educational posts, informational videos, and professional updates related to healthcare topics, as well as personal content such as selfies, social gatherings, and vacation scenes. We verified that all materials were following local regulations governing medical content and online communication. And the recruited patients are those who visit the hospital for in-person consultations. The recruitment criteria included: individuals aged between 18 and 65, those with a scheduled follow-up appointment within 1–2 months, and participants willing to complete an anonymous survey about social media usage and treatment outcomes.

The data collection procedures are as follows. Initially, the 50 doctors were asked to provide their demographic information, including age, gender, education level, and professional title. They also completed a questionnaire regarding their social media behavior and humor. The questionnaire was designed to assess various aspects of their social media behavior and humor. It included questions on the types of content they create and share, the frequency of their posts, and their engagement with followers, as well as questions aimed at evaluating the use of humor in their interactions with patients. Subsequently, in the outpatient department, with the help of doctors, the research team selected patients who met the following criteria. The participants were informed that the survey was voluntary and would not impact their medical care. After receiving verbal consent from the patients, they were invited to follow one of the doctor’s social media channels and asked to complete a paper-based questionnaire. This questionnaire gathered demographic information, including age, gender, and education, as well as specific details such as financial pressure, duration of the visit, number of visits to the doctor, and general health status. Once completed, the research team collected the questionnaires and assigned them sequential numbers based on the order of treatment. Finally, once the patients’ treatment was completed, either entirely or partially (for those undergoing phased treatment lasting over 2 years), the doctors individually evaluated the patients’ adherence to treatment and the treatment outcome. It is important to emphasize that we assure all doctors and patients that the collected data will be completely confidential and used only for scientific research.

### 2.2 Participants

After statistics, we successfully collected a total of 45 doctors’ self-assessment questionnaires, 378 patient basic information questionnaires, and 337 doctors evaluating patient adherence and treatment outcome questionnaires. After screening incomplete, inaccurate, and unmatched questionnaires, 35 doctors’ self-assessment questionnaires, 322 patient questionnaires, and 322 questionnaires evaluating patients from doctors were used for data analysis.

Regarding the doctors, the mean age is 37.65 years old (SD = 7.21), with 37.1% male and 62.9% female. They have a high level of education, with 54.3% holding a master’s degree and 25.7% holding a doctoral degree, and over half of them (54.3%) attending doctor. As for the patients, their average age is 32.86 years old (SD = 13.05). Among them, 41.6% are male and 58.1% are female. Over half of them (51.4%) have at least a bachelor’s degree. Additionally, 70% of the patients have visited their doctors two times or more.

### 2.3 Measures

The measures in this study were adapted from established scales, and all survey items were evaluated using a 5-point Likert scale (ranging from 1 = “strongly disagree” to 5 = “strongly agree” or ranging from 1 = “never” to 5 = “always”). All items were initially drafted in English and then translated into Chinese using the back-translation procedure. We conducted a reliability analysis for each scale used in our survey to ensure that the measures were internally consistent within our sample. The Cronbach’s alpha values reported are specific to this study, reflecting the reliability of the instruments as applied in our research context.

#### 2.3.1 Social media behavior

We categorize doctor social media behavior into two types: professional utilization and personal utilization (7, 27). Doctors self-rated the frequency of posting different types of content on social media. The subscale for professional utilization consists of 3 items, with a sample item being: “Sharing the latest research findings” (*α* = 0.85). The subscale for personal utilization consists of 4 items, with a sample item being: “Sharing family photos” (*α* = 0.85).

#### 2.3.2 Doctor humor

Doctor humor was measured with a 3-item scale developed by Cooper et al. (24). Doctors self-rated the frequency of expressing humor when interacting with patients. Sample items included: “How frequently do you express humor with your patient at work, overall?.” The Cronbach’s *α* for the 3-item scale is 0.92.

#### 2.3.3 Patient adherence

Patient adherence was assessed by doctors’ objective evaluation of patients’ performance in general adherence, medication adherence, exercise adherence, and diet adherence (28). This approach was adopted because self-report methods are susceptible to overestimating compliance and underestimating non-compliance (17). The Cronbach’s *α* for the 4-item scale is 0.82.

#### 2.3.4 Treatment outcome

After treatment, doctors assess the stability, aesthetics, ability to chew, and ease of speaking of the patients’ teeth (29). Additionally, there is another item to evaluate the overall treatment outcome. The Cronbach’s *α* for the 5-item scale is 0.88.

#### 2.3.5 Control variables

We included demographic information of doctors and patients as control variables. Specifically, these variables included age, gender, education level, and professional title. Additionally, we considered factors such as patient financial pressure, duration of the visit, number of visits to the doctor, and general health status of the patient, which can influence the doctor-patient relationship, patient adherence, and treatment outcome (18, 30). Financial pressure was included as a variable in our study due to its potential impact on patient behavior and treatment outcomes. Patients facing financial stress might prioritize immediate financial needs over long-term healthcare, which may affect their willingness and ability to adhere to prescribed treatments or attend follow-up appointments. This is especially relevant in the context of specialized dental care, where costs can be significant.

## 3 Results

### 3.1 Common method bias and confirmatory factor analyses

To alleviate the impact of common method bias, data was collected at two separate time points. Harman’s single factor procedure was then applied to address this issue (31). The exploratory factor analysis (EFA) was conducted using SPSS version 25.0, revealing five factors with eigenvalues greater than 1, with the first factor explaining 26.64% of the total variance—significantly below the critical 40% threshold. Hence, common method variance is unlikely to be a serious problem in our case.

Next, we used Mplus 8.0 to conduct confirmatory factor analysis (CFA), comparing the five-factor model with alternative models to assess the distinctiveness of the key variables. As shown in Table 1, the five-factor model fits the data significantly better than the alternatives [*χ*^2^(24) = 36.21, *p* < 0.001; CFI = 0.99, TLI = 0.99, RMSEA = 0.04, SRMR = 0.03].

**Table 1:** Results of confirmatory factor analyses.

Model	*χ* ^2^	*df*	TLI	CFI	RMSEA	SRMR
Five-factor model	36.21	24	0.99	0.99	0.04	0.03
Four-factor model^a^	387.10	29	0.67	0.788	0.20	0.12
Three-factor model^b^	721.67	32	0.42	0.59	0.26	0.14
Two-factor model^c^	826.93	34	0.38	0.53	0.27	0.16
One-factor model^d^	3101.38	152	0.20	0.29	0.25	0.19

^aProf U and Pers U combined; bDC, PA, and TO combined; cProf U and Pers U combined, DC, PA, and TO combined; dProf U, Pers U, DC, PA, and TO combined. Prof U is professional utilization of social media, Pers U is personal utilization of social media, DH is doctor humor, PA is patient adherence; TO is treatment outcome. TLI is the Tucker–Lewis index, CFI is the comparative fit index, RMSEA is the root-mean-square error of approximation, and SRMR is the standardized root mean square residual.^

### 3.2 Descriptive statistics headings

The means, standard deviations, and correlations among the research variables are presented in Table 2. As the table shows, personal utilization is negatively correlated with patient adherence (*r* = −0.17, *p* < 0.01) and treatment outcome (*r* = −0.11, *p* < 0.05), whereas professional utilization is positively correlated with patient adherence (*r* = 0.22, *p* < 0.01) and treatment outcome (*r* = 0.11, *p* < 0.05). Additionally, patient adherence is positively correlated with treatment outcome (*r* = 0.58, *p* < 0.01).

**Table 2:** Means, standard deviations, and bivariate correlations of variable.

Variables	Mean	SD	1	2	3	4	5	6	7	8	9	10	11	12	13	14	15	16
1. D-Age	37.65	7. 21	1															
2. D-Edu	4.09	0.66	0.23**	1														
3. D-PT	2.30	0.74	0.67**	0.67**	1													
4. P-Age	32.86	13.05	−0.03	0.07	−0.02	1												
5. P-Gen	–	–	0.07	0.09	0.11*	−0.22**	1											
6. P-Edu	3.05	0.87	−0.03	−0.06	−0.04	−0.30**	0.00	1										
7. P-FP	2.23	1.03	−0.00	0.08	0.02	0.02	0.05	−0.08	1									
8. DUR	2.44	5.02	−0.05	−0.00	−0.02	−0.02	0.05	0.05	−0.07	1								
9. Num	3.14	4.20	0.20**	−0.18**	0.07	−0.06	0.04	0.06	−0.21**	0.00	1							
10. PHS	3.97	0.87	−0.10**	−0.03	−0.02	−0.28**	0.09*	0.18**	−0.25**	0.06	0.13*	1						
11. Pers U	1.80	0.72	0.25**	−0.17**	0.15**	0.03	0.03	0.07	0.03	0.03	−0.08	0.06	(0.79)					
12. Prof U	2.84	0.85	0.11*	0.18**	0.10*	0.06	0.08	−0.03	0.17**	−0.07	−0.09*	−0.02	0.20**	(0.84)				
13. D-Gen	–	–	−0.11*	0.17**	0.23**	−0.07	0.02	−0.06	0.00	−0.07	0.07	0.04	−0.17**	−0.12*	–			
14. DH	2.72	0.84	0.21**	0.17**	0.18**	0.05	−0.08	−0.03	0.05	0.03	−0.07	−0.13*	−0.02	0.32**	−0.04	(0.90)		
15. PA	4.26	0.58	−0.07	0.08	−0.12*	−0.05	−0.02	0.02	−0.00	0.08	0.03	0.10*	−0.17**	0.22**	−0.22**	0.01	(0.73)	
16. TO	4.42	0.52	−0.13*	0.12*	−0.01	−0.08	0.02	0.04	−0.06	0.08	0.02	0.14**	−0.11*	0.11*	−0.27**	0.01	0.58**	(0.78)

*p < 0.05, **p < 0.01. The diagonal elements in the parentheses represent the square root of AVE. P-FP is patient financial pressure; DUR is duration of the visit; NUM is the number of visits to the doctor; PHS is patient general health status.

### 3.3 Psychometric properties

Table 3 shows the evaluation of composite reliabilities and convergent validity. The composite reliabilities (CR) exceed the 0.7 threshold, demonstrating high reliability for all constructs. Additionally, the average variance extracted (AVE) surpasses the threshold value of 0.5, satisfying the criteria for convergent validity. Discriminant validity is evaluated by comparing the square root of the AVE with the correlations between each construct and others (32). As shown in Table 2, the square root of the AVE for each construct is greater than its correlations with other constructs, thereby confirming the discriminant validity of the five constructs.

**Table 3:** Psychometric properties of the measurement model.

Variables	AVE	CR
Pers U	0.62	0.86
Prof U	0.71	0.87
Doctor humor	0.81	0.93
Patient adherence	0.54	0.82
Treatment outcome	0.60	0.88

CR is composite reliability; AVE is average variance extracted.

### 3.4 Main analyses

**Figure 1:**
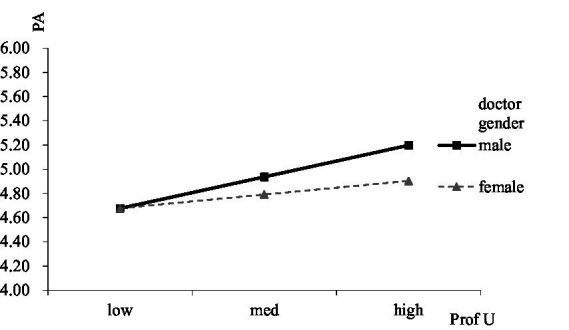
Standardized path coefficients. ** p < 0.01, *** p < 0.001.

We used Structural equation modeling (SEM) in Mplus 8.0 software to identify doctors’ social media behavior, patient adherence, treatment outcome, doctor gender, and doctor humor. The research model with standardized maximum likelihood estimates for path coefficients is presented in Figure 1. The overall model showed a good fit (*χ*^2^ = 4.38, df = 3; CFI = 0.99, TLI = 0.97, RMSEA = 0.04, SRMR = 0.01). According to the structural equation model (Figure 1), the professional utilization of social media showed a positive and significant relationship with patient adherence (*β* = 0.17, s.e. = 0.06, *p* < 0.01), while personal utilization is negatively related to patient adherence (*β* = −0.24, s.e. = 0.05, *p* < 0.001). Patient adherence was positively related to treatment outcome (β = 0.35, s.e. = 0.04, *p* < 0.001). The total indirect effect of professional utilization on treatment outcome was significantly positive (*β* = 0.07, s.e. = 0.03, *p* < 0.01). While the total indirect effect of personal utilization on treatment outcome was significantly negative (*β* = −0.11, s.e. = 0.03, *p* < 0.001). Thus, patient adherence mediates the relationship between professional utilization and treatment outcome as well as the relationship between personal utilization and treatment outcome.

**Figure 2:**
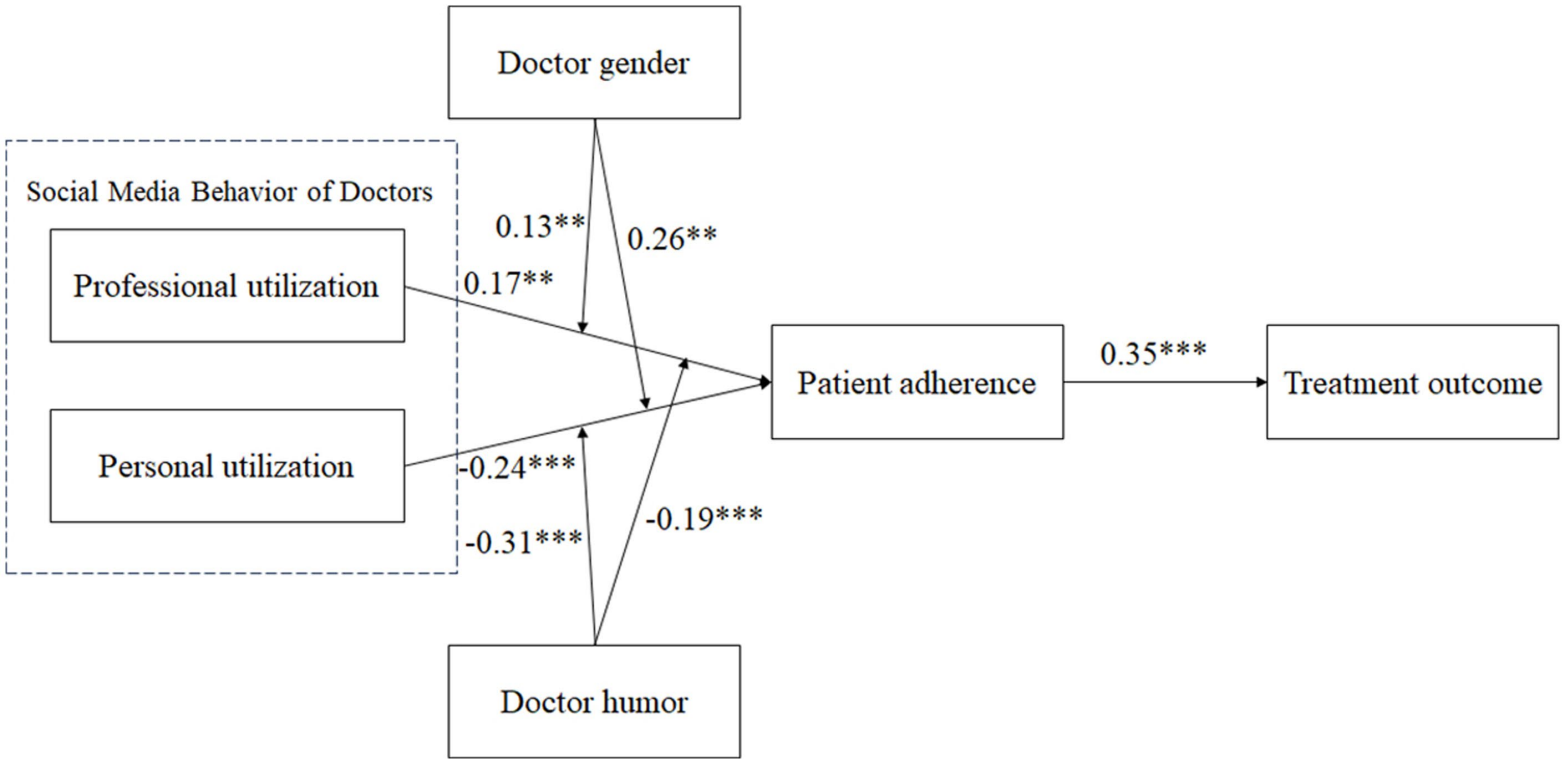
The moderating effect of doctor gender on the relationship between professional utilization of social media by doctors and patient adherence.

**Figure 3:**
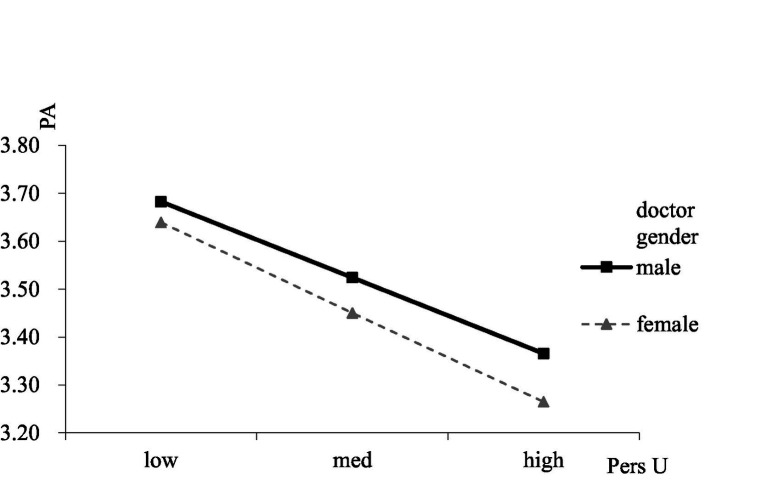
The moderating effect of doctor gender on the relationship between personal utilization of social media by doctors and patient adherence.

**Figure 4:**
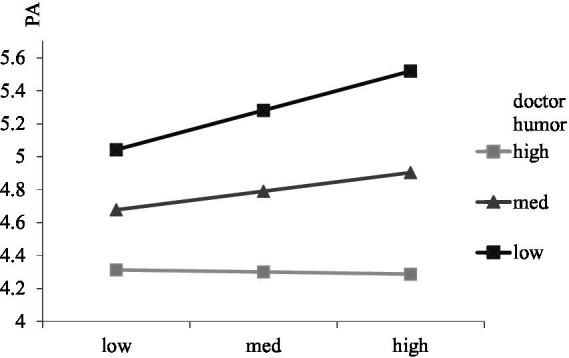
The moderating effect of doctor humor on the relationship between professional utilization of social media by doctors and patient adherence.

**Figure 5:**
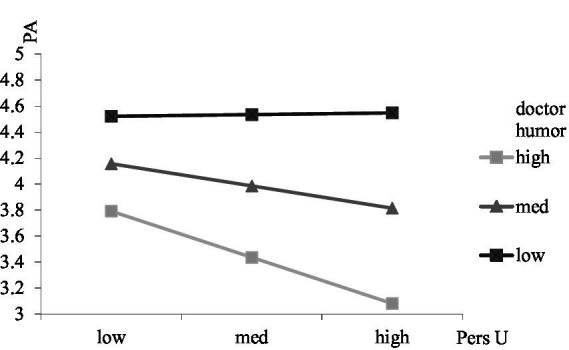
The moderating effect of doctor humor on the relationship between personal utilization of social media by doctors and patient adherence.

To test the moderating roles of doctor gender and humor, interactions between professional utilization and doctor gender, personal utilization and doctor gender, professional utilization and doctor humor, and personal utilization and doctor humor were calculated. The result showed that the interaction between professional utilization and doctor gender was significantly related to patient adherence (*β* = 0.13, s.e. = 0.06, *p* < 0.05), and the positive relationship between professional utilization and patient adherence was stronger for men (*β* = 0.14, s.e. = 0.07, *p* < 0.05) than women (*β* = 0.13, s.e. = 0.06, *p* < 0.05; see Figure 2). The interaction between personal utilization and doctor gender was significantly related to patient adherence (*β* = 0.26, s.e. = 0.06, *p* < 0.001), and the negative relationship between personal utilization and patient adherence was stronger for women (*β* = −0.26, s.e. = 0.05, *p* < 0.001) than men (*β* = −0.22, s.e. = 0.05, *p* < 0.05; see Figure 3). The result showed that the interaction between professional utilization and doctor humor was significantly related to patient adherence (*β* = −0.19, s.e. = 0.05, *p* < 0.001), and the positive relationship between professional utilization and patient adherence was stronger when doctor humor was low (M-SD; *β* = 0.424, s.e. = 0.07, *p* < 0.001) rather than high (M + SD; *β* = 0.068, s.e. = 0.06, *p* > 0.05; see Figure 4). The interaction between personal utilization and doctor humor was significantly related to patient adherence (*β* = −0.31, s.e. = 0.05, *p* < 0.001), and the negative relationship between personal utilization and patient adherence was stronger when doctor humor was high (M + SD; *β* = −0.22, s.e. = 0.05, *p* < 0.001) rather than low (M-SD; *β* = −0.03, s.e. = 0.05, *p* > 0.05; see Figure 5).

## 4 Discussion

Based on SEM, this study explored the impact of doctors’ social media behavior (professional utilization and personal utilization) on patient adherence and treatment outcome, as well as the moderating role of doctor gender and doctor humor. We meticulously evaluated the potential for content shared by doctors on social media to exert a direct and significant influence on patients’ health behaviors and outcomes, with the possibility of yielding contrasting results in certain instances. The empirical findings of this study provide valuable insights into the doctor-patient interaction in social media context.

First, the research findings revealed a positive relationship between doctors’ professional use of social media and patient adherence and treatment outcomes, while their personal use of social media has a negative correlation with patient adherence and treatment outcomes. The content posted by doctors on social media is inevitably exposed to patients, regardless of the purpose. This exposure has the potential to influence how patients perceive and judge the doctors, ultimately impacting the doctor-patient relationship and actual health outcomes. The finding is supported by the role literature indicating that role stereotypes and corresponding expectations, such as gender roles and occupational roles, are relatively fixed and influence the behavior of certain groups and the responses of observers (33). In the medical context, doctors, as a profession, are commonly associated with occupational stereotypes such as being intelligent, powerful, competent, and warm, but unemotional (34–36). Thus, when doctors post professional knowledge content on social media, it is congruent with patients’ expectations for their role as doctors, thus increasing patient adherence and treatment effectiveness. Conversely, when doctors shared personal life-related content on social media, patients perceived the behavior as unprofessional, resulting in reluctance to follow the doctors’ advice and negative effects on treatment outcome. The findings also align with previous research on the effects of doctors’ social media behavior. For example, Wu et al. (2) found that doctor-consumer interaction on social media would influence consumers’ health behaviors. Fatollahi et al. (10) found that if the physician posts a respectful narrative on social media, around one-third (35%) of patients reported an increase in trust in their physician. Conversely, if the provider writes a disrespectful patient narrative on social media, 84% of patients would decrease their trust in the provider. Our study builds on these findings, proposing that patients might evaluate their doctors based on their social media behavior—both professional and personal—which may impact health behaviors and outcomes.

Second, the study examined the moderating effects of doctor gender on the relationship between doctor social media behavior and patient adherence. Consistent with previous research on gender stereotypes (37), the results indicated that female doctors engaged in professional work may encounter gender bias. Compared to male doctors, female doctors had a weaker positive impact on patient adherence when posting professional knowledge on social media, but a stronger negative impact when sharing personal life-related content on social media. This is consistent with prevalent gender stereotypes in society, which elicit different patient reactions to similar social media behavior exhibited by male and female doctors. Especially in China, women are generally expected to take on more nurturing, caregiving, and family-oriented roles, while men are seen as more suited for homemaking, professional, and leadership roles (38). Historically, physicianhood has been conceived as “masculine” (39), with male doctors perceived as more authoritative and professional (23), while female doctors receive less respect than their male colleagues (19). In the context of Chinese culture, this study explored patients’ responses to the social media behaviors of doctors based on their gender, thereby further enriching the literature on gender bias, doctors’ social media behaviors, and their social effects.

Third, the results showed that doctors’ humor also influenced patients’ perception and behavior, weakening the positive impact of doctors’ professional utilization on patient adherence and enhancing the negative impact of personal utilization on patient adherence. Scholars and practitioners have argued that humor is a valuable tool for enhancing the doctor-patient relationship (40). Empirical studies have consistently demonstrated that a perceived sense of humor from doctors is positively linked to their credibility, patient satisfaction, and patient compliance (26). However, under certain circumstances, displaying humor inappropriately may be less effective or even harmful (40, 41), particularly in remote communication on social media platforms, where misunderstandings can occur quickly. What is more, in China, a notable cultural aspect is the significant reverence people hold for doctors, who are often referred to as “angels in white” (36). Patients generally expect doctors to demonstrate empathy toward their suffering and communicate in a serious and professional manner (42). Thus, the tension between doctors’ sense of humor and their professional role may undermine patients’ trust and perception of doctors who share content on social media (12). While prior research has explored the multiple effects of doctor humor, this study focused on the unique role and impact of humor in the context of Chinese culture and the online environment, thereby enhancing the understanding of humor across different contexts.

### 4.1 Research implications

This research contributes to the literature on social media behaviors by investigating how various social media behaviors of doctors influence patient adherence and overall health outcomes. While previous studies have examined the impact of doctors’ social media behavior on doctor-patient trust (10), the influence of such behavior on actual treatment outcomes remains unknown. Compared to previous research, the current study extends the investigation of doctors’ social media behavior to include its effects on patients’ adherence behaviors and health outcomes. Furthermore, this study examines the moderating effects of doctor gender and humor on the relationship between doctor social media behavior and patient adherence, empirically clarifying the boundary conditions of social media behavior.

This study also enriches the study of patient adherence antecedents and outcomes by examining the influence of doctor social media behavior, gender, and humor. Prior studies have primarily focused on factors related to patient adherence, including patient-doctor communication, treatment regimens, patient characteristics, and family support (17, 30, 43). However, this study introduces a novel perspective by incorporating doctor social media behavior and personality traits into the analysis, emphasizing the interactions among these variables and their potential impact on patient adherence. Moreover, the impact of patient adherence on treatment outcomes has been validated in a sample of patients from China (18).

This study has key practical implications for doctors and healthcare organizations to improve the doctor-patient relationship and promote the therapeutic effect. First, the research findings indicate that both the professional and personal use of social media by doctors can significantly impact patient adherence and treatment outcomes, but the effect is notably distinct. On the one hand, doctors use social media to gather and share treatment information (7), promote their research (44), and communicate with patients (8). Additionally, social media provide doctors a means to engage in leisure activities (45), such as sharing life news, connecting with friends (7), or even posting derogatory speech and alcohol images (46). Therefore, it is essential for doctors to uphold appropriate professional boundaries (13), differentiate between personal and professional social media accounts, and remain cognizant of the potential impact on patient health outcomes prior to posting unprofessional content on their public social media accounts.

Second, our findings revealed that female doctors are more likely to face negative reactions from social perceivers. Thus, female doctors should be more alert and cautious when using public social media and emphasize the depth and practicality of their content. For example, by incorporating case studies and analyses of real-life examples, they can enhance the appeal and persuasiveness of their communications, thus fostering increased patient trust and adherence. At the same time, it became evident that humor intensifies the negative behavioral reactions from patients. When sharing professional medical information on social media platforms, it is essential for doctors to maintain a serious and accurate tone and to avoid the use of humor that may be ambiguous or misleading, especially in China.

Last, healthcare organizations should provide clear guidelines and strategies to navigate appropriate social media use in the digital age. Doctors should be thoroughly informed about the potential consequences of their online actions, including how to uphold confidentiality, respect patient privacy, and avoid any behavior or content that could jeopardize the doctor-patient relationship. In practice, healthcare organizations can establish official social media accounts to serve as primary platforms for disseminating authoritative medical information and addressing patient inquiries, which can reduce patient confusion and misunderstandings from mixed information, ultimately improving patient adherence. It is also necessary to develop and enforce comprehensive ethical guidelines for the creation and deployment of AI-based personas in healthcare social media contexts. These guidelines should address issues such as data privacy, consent, algorithmic bias, and the potential for manipulation or misrepresentation of information.

### 4.2 Limitations and future research

Despite the contributions of our study, it is important to acknowledge several limitations. First, since the primary focus of this study is on how different social media behavior of physicians impact patient adherence and treatment outcomes, our research does not currently address the role of key opinion leaders (KOLs). According to the two-step flow of communication theory, opinion leaders carefully analyze mass media content and then convey their interpretations to a broader audience (47). In medicine, KOLs include both physicians and non-physician scientists who are hired by pharmaceutical companies as consultants and to influence medical practices, such as prescribing habits and treatment contributions (48). Therefore, the presence of KOLs on social media could potentially amplify or alter the effects identified in our study. Future research could further examine how the presence and behavior of KOLs on social media might affect patient adherence and treatment outcomes differently compared to regular healthcare professionals.

Second, our study only considered the moderating effect of doctors’ gender, neglecting other potential characteristics that could influence patients’ reactions to doctor social media behavior. For example, Surani et al. (45) discovered that younger healthcare professionals tend to utilize social media more frequently than their older counterparts. Moreover, it is important to note the significant variations in individual reactions to social media postings. Therefore, future research should encompass patients’ characteristics to comprehensively examine the various factors that may impact the relationship between doctor social media behavior and patient outcomes.

Third, although this research indicated that humor displayed by doctors hinders patient adherence, it is essential to acknowledge that this conclusion may not be all-encompassing. It is worth mentioning that humor researchers have categorized interpersonal humor into affiliative humor and aggressive humor (49). Thus, exploring the effects of both positive and negative humor expressions by doctors on the doctor-patient relationship would provide an intriguing direction for future research.

Fourth, the sample data was only derived from Chinese dentists and patients, and there may be variations in role expectations and stereotypes for both doctors and patients due to geographical and cultural differences. Therefore, future studies should consider expanding the sample to include more diverse populations to assess the generalizability of our findings.

## 5 Conclusion

In conclusion, this study provides empirical evidence on how doctor social media behavior influences patient adherence and treatment outcomes, highlighting the moderating effects of doctor gender and humor. By utilizing a diverse sample of healthcare professionals and patients, the study provided comprehensive coverage of its objectives, capturing a broad spectrum of data on social media behavior and its effects on patient interactions. The study offered a thorough examination of how social media behavior affects patient outcomes across diverse demographics and personality types. The insights gained from this study offer valuable guidance for doctors on optimizing their social media practices and communication strategies, ultimately aiming to improve patient adherence and treatment effectiveness.

## Data Availability

The raw data supporting the conclusions of this article will be made available by the authors, without undue reservation.
